# Comparative Study of DTMUV and LPS on Duck Liver Disease

**DOI:** 10.3390/vetsci12090900

**Published:** 2025-09-17

**Authors:** Zhenghui Lan, Zhigang Sun, Yi Wang, Huatao Li, Xuejing Sun

**Affiliations:** College of Veterinary Medicine, Qingdao Agricultural University, Qingdao 266109, China; zhenghuil0429@163.com (Z.L.); sl612429@163.com (Z.S.); 19932727671@163.com (Y.W.); huataoli@foxmail.com (H.L.)

**Keywords:** duck, liver, duck tembusu virus, lipopolysaccharide, comparative histopathology

## Abstract

This study investigated the structural features of adult duck liver and compared pathological alterations induced by duck Tembusu virus (DTMUV, strain XZ-2012) and lipopolysaccharide (LPS). Histological techniques (HE, reticular fiber, and trichrome staining) revealed normal duck liver exhibited reddish-brown coloration with indistinct lobule boundaries and no prominent bile ducts. Kupffer cell distribution was mapped via jugular ink injection. DTMUV infection caused liver swelling, congestion, and yellowish discoloration. Histopathology showed lymphocyte infiltration around central veins and portal areas, increased reticular fibers, thickened basement membranes, hepatocyte vacuolation, and erythrocyte accumulation in sinusoids. In contrast, LPS exposure led to mild hepatic enlargement without vacuolar degeneration but with marked perivascular lymphocyte aggregation and reticular fiber proliferation. Both treatments elevated Kupffer cell numbers. These findings demonstrate distinct liver injury patterns: DTMUV induces direct hepatocellular damage with inflammatory responses, while LPS triggers intense immune cell recruitment without significant hepatocyte degeneration. The study provides insights into avian viral versus bacterial pathogenesis and liver defense mechanisms, offering a foundation for further research into waterfowl infectious diseases.

## Simple Summary

This study examined the structural characteristics of adult duck liver and compared pathological changes induced by duck Tembusu virus (DTMUV, strain XZ-2012) and lipopolysaccharide (LPS). The normal liver appeared reddish-brown with indistinct lobule boundaries and no prominent bile ducts. Kupffer cell distribution was mapped via jugular ink injection. DTMUV infection caused swelling, congestion, and yellowish discoloration, with histopathology showing lymphocyte infiltration around central veins and portal areas, increased reticular fibers, thickened basement membranes, hepatocyte vacuolation, and erythrocyte accumulation in sinusoids. In contrast, LPS exposure produced only mild hepatic enlargement without vacuolar degeneration but with pronounced perivascular lymphocyte aggregation and reticular fiber proliferation. Both treatments increased Kupffer cell numbers. These findings reveal distinct patterns of liver injury: DTMUV primarily induces direct hepatocellular damage accompanied by inflammation, while LPS mainly triggers strong immune cell recruitment without significant hepatocyte degeneration. The results provide valuable insights into differences between viral and bacterial liver pathogenesis in waterfowl, offering a basis for further studies on infectious disease mechanisms.

## 1. Introduction

Duck Tembusu virus (DTMUV) belongs to the Flaviviridae family, a single-stranded positive-stranded RNA virus [1], which can infect birds, especially meat ducks and egg ducks. The infection rate is 100%, and the elimination rate is very high. DTMUV disease is not affected by seasons. It is a viral acute infectious disease, which will lead to a decline in feed intake and egg production, or even a loss of production [2]. It will develop slowly, and some will be accompanied by neurological symptoms. It has become one of the most serious infectious diseases in the current duck industry, and has seriously affected the economic benefits of the industry. Anatomically, it was found that the female duck had ovarian hemorrhage and necrosis, followed by splenomegaly, marble-like necrosis, liver edema, yellowing and bleeding, and multiple organs, such as the brain, pancreas, heart, and intestine, would produce different levels of pathological changes [3]. In the early studies, there have been further studies on spleen diseases [4,5]. However, limited research has been conducted on the effect of DTMUV on the liver. Notably, DTMUV infection induces hallmark pathological changes in viral hepatitis in the liver. The manifestations include hepatocellular vacuolar degeneration, focal necrosis, inflammatory cell infiltration, and potentially bile duct hyperplasia with fibrosis [6,7].

Infections caused by multiple Gram-negative bacteria have become a global threat to the duck feed industry, hindering the sustainable development of poultry. Lipopolysaccharide (LPS) is considered to be the most important molecule responsible for the pathological process of infection with a variety of Gram-negative bacteria [8]. LPS, also known as “endotoxin”, is the main component of the cell wall of Gram-negative bacteria, mainly composed of core polysaccharide, O-polysaccharide side chain, and lipoid A, of which lipoid A is the main toxic component of Gram-negative bacteria [9]. LPS is associated with various inflammatory and immune responses and threatens the health of livestock and poultry [10]. In the process of infection and disease induction, LPS can increase the immune capacity of the body by activating the mononuclear macrophage system to inhibit the damage of pathogenic microorganisms to the body [11]. LPS-induced liver morphology was disrupted, with markedly swollen hepatocytes and loss of cytoplasmic contents. Simultaneously, the hepatic lobule structure was disorganized, sinusoids were difficult to identify, and were accompanied by extensive inflammatory cell infiltration [12,13].

The liver is an important and complex organ of the body. It can synthesize plasma protein, lipoprotein, cholesterol, bile salt, glycogen, and other important substances in the body, secrete bile, detoxification functions, storage of glycogen, vitamins, and trace elements, as well as participate in the body’s defense mechanism [14]. During the fetal period, the liver is an important hematopoietic organ. The liver has a significant number of Kupffer cells, which ingest the majority of harmful microbes that invade the liver. Hepatic macrophages have a powerful antibacterial and viral effect, bind to pathogens or phagocytose pathogens for immune recognition, mediate the inflammatory response of liver tissue, inhibit the replication of pathogenic microorganisms in liver cells, activate the immune response of neighboring cells, activate or attract related immune cells, and interact with them to further complete the antiviral and inflammatory response [15]. Although the liver of birds is similar to mammals in cell composition, its structure is different. The damage of DTMUV to reproductive organs and immune organs is more significant, but there are few studies on the pathological changes in digestive glands, such as the liver. Some studies have found that in natural infection cases, the liver of the most severely diseased duck has obvious damage, and it is speculated that liver failure is a major cause of death [16]. In this experiment, we studied the structure of adult duck liver by morphological methods and compared the liver lesions caused by two different antigens of DTMUV and LPS, providing a theoretical basis for the pathogenicity of different antigens to birds and the defense mechanism of the liver.

## 2. Methods

### 2.1. Experimental Animals

Healthy four-month-old female ducks were purchased from Taihe Farm in Ji’an, Jiangxi Province, with an average weight of about 1.5 kg. SPF duck embryo was purchased from Shandong Haotai Experimental Animal Breeding Co. (Jinan, China). Purified allantoic fluid DTMUV XZ-2012 was presented by the Laboratory of Cell and Developmental Biology, School of Animal Medicine, Nanjing Agricultural University.

### 2.2. Preparation of Injection

The purchased SPF duck embryos were incubated in a thermostatic incubator until the age of 10 days, and DTMUV was inoculated in the allantoic cavity (200 each μL). Dead SPF duck embryos must be processed within 24 h. Based on preliminary data from previous avian models, lesions are most prominent at the 3-day time point; therefore, allantoic fluid is collected after 3 days.

EID50 (half infective dose of duck embryo): The seed virus was diluted 10 times using sterile normal saline, inoculated into 11-day-old duck embryo through the allantoic cavity, with 0.2 ml of each embryo. At the same time, the normal saline was used as the control, and cultured at 37 °C. The eggs were continuously observed for 7 days, and the duck embryos that died within 24 h were discarded. After 7 days, the dead and live duck embryos were collected. The results were observed and recorded. Allantoic fluid was collected after incubation, followed by RT-PCR assay. EID 50 values of positive samples were calculated using the Reed–Muench method.

Virus injected intramuscularly 1.5 × 10^4^ EID50, lipopolysaccharide was diluted to 10 mg/mL with sterile physiological saline, and injected intraperitoneally at 5 mg/kg, ink was diluted to 10% with sterile physiological saline, and injected intravenously at 8 mL.

### 2.3. Sample Collection

A total of 18 ducks were selected for this experiment, 6 in the control group, 6 in the DTMUV-infected group, and 6 in the LPS-inoculated group. The ducks were executed by decapitation 1 d after virus infection and 3 h after lipopolysaccharide inoculation, and half of the ducks in each group were injected with 2% 8 mL of ink 1 h before execution. The material was taken after dissection, and the liver tissues were washed with 0.02 M PBS, then divided into tissue blocks of appropriate size, placed in 4% paraformaldehyde fixative for 48 h, and made conventional paraffin-embedded sections. All animal experiments were approved by the Animal Ethics Committee of Qingdao Agricultural University. For the animal experiments, all efforts were taken to minimize the suffering of the animals, all methods carried out in the study were performed in accordance with relevant guidelines and regulations.

### 2.4. HE Staining

The thickness of the paraffin section is 6 μm. Then, conventional dewaxing, Haematoxylin and eosin staining, and dehydration were carried out, and finally, neutral resin film was sealed and observed with an optical microscope.

### 2.5. Reticulate Fiber Staining

The paraffin sections (The thickness of the paraffin section is 12 μm) were assessed by the silver impregnation method specified by Gordon and Sweets (Gordon and Sweets, 1936). Briefly, deparaffinized sections were rinsed in distilled water between each incubation step of 1% acidified potassium permanganate for 5 min, 1% oxalic acid for 2 min, 2.5% iron alum for 15 min, ammoniacal silver solution for 5 min, 10% aqueous formalin for 2 min, 0.2% gold chloride for 2 min, and 5% sodium thiosulfate for 4 min, and the sections were then dehydrated and sealed by neutral gum piece.

### 2.6. Trichrome Staining with Masson Trichrome Staining Kit

It was used to stain collagen fibers, reticular fibers, and other connective tissues. Following the steps in the kit instructions for staining, collagen fibers can be stained blue, and cytoplasm is stained red.

## 3. Results

### 3.1. Anatomy and Structure of Duck Liver

**Figure 1:**
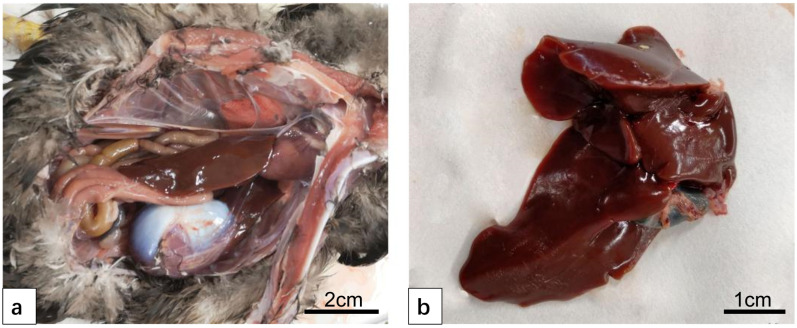
The anatomical structure of the normal duck liver. (**a**) Duck liver appears reddish-brown in appearance. (**b**) The liver is divided into two lobes, the right lobe is larger and is connected to the gallbladder.

The liver was a large, normally reddish-brown liver located in the abdominal cavity. The heart and pericardium were clamped in front of the liver, and the glandular stomach and muscular stomach were clamped in the back of the liver (Figure 1a).

The liver was divided into two lobes, a left lobe and a right lobe. The left and right lobes were connected to each other through the isthmus. The right lobe was slightly larger than the left lobe. There was a hepatic portal in the left and right lobes, and there were hepatic arteries, portal veins, and hepatic ducts in and out of the hepatic portal. The gallbladder communicates with the hepatic duct of the right lobe, not with the hepatic duct of the left lobe, but with the gallbladder duct opens at the end of the duodenum. The liver had a serous membrane on the surface and parenchyma on the inside. Clearly thickened connective tissue can be seen at the hilum of the liver. The connective tissue enters from the hilum of the liver and divides the liver parenchyma into many lobules (Figure 1b).

**Figure 2:**
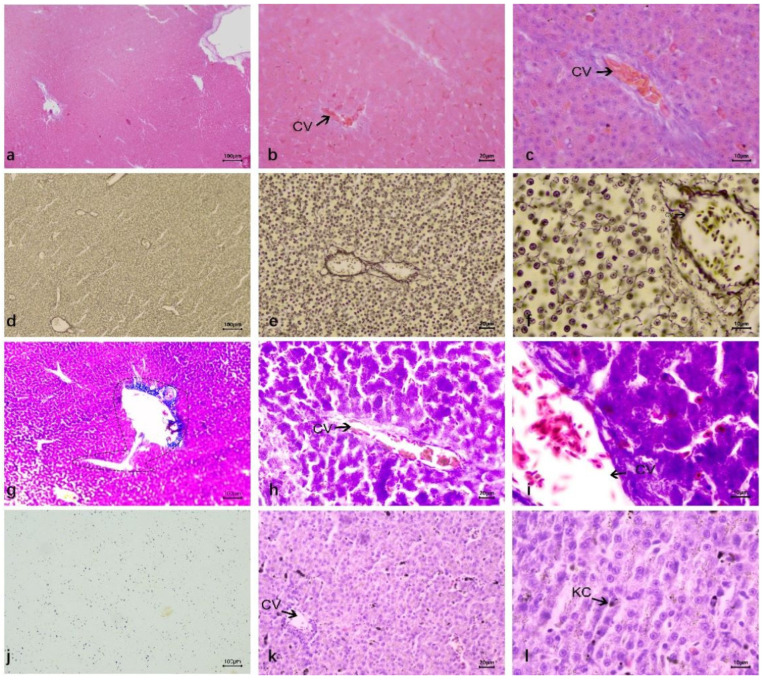
The histological structure of normal duck livers. (**a**–**c**) The HE staining results. (**a**) Duck liver: underdeveloped interlobular connective tissue (indistinct lobule boundaries); lobules consist of central vein, hepatic plate, and sinusoids. (**b**) Hepatic lobule center: thin-walled, narrow central vein (single-layer endothelial cells). Surrounding radial hepatic plates (hepatocytes); duck hepatic plates are special: two rows of parallel hepatocytes with anastomosing branches forming a reticular structure. Hepatic sinusoids (endothelial cells) between plates open into the central vein. (**c**) Duck hepatocytes: polyhedral with clear outlines; round, large nuclei (mostly central, few binucleate), lightly stained with clear envelopes and 1–2 nucleoli each. (**d**–**f**) Reticular fiber staining results. (**g**–**i**) Masson trichrome staining results. Reticular fibers in the central vein basement membrane, interlobular vessels, and hepatic sinuses (discontinuous basement membrane); no radial fibers around the central vein or vascular structures between hepatocytes. (**j**–**l**) Dyeing results after carbon injection. Abundant carbon granules in hepatic sinuses, phagocytosed by KC; Central vein (CV); Kupffer cells (KC).

In HE staining, the liver structure of ducks was different from that of other mammals. The connective tissue between the lobules was underdeveloped, with indistinct lobule boundaries. However, the basic structure of the lobules was relatively clear, including the central vein, hepatic plate (hepatic cord), and hepatic sinusoids (Figure 2a). The middle part of the hepatic lobule is the central vein, which consists of a layer of endothelial cells, forming a relatively thin vascular wall with a narrow tube diameter. Under the microscope, interlobular arteries and interlobular veins were distributed in the portal duct area between the hepatic lobules of ducks, both of which ran in parallel. The wall of the interlobular artery had obvious smooth muscle, which was thicker than the wall of the interlobular vein, and the lumen was smaller than that of the interlobular vein; no obvious interlobular bile duct was found. The entrance and exit of the hepatic sinuses can be clearly seen around the central vein is a liver plate composed of liver cells arranged radially. The duck liver plate is special, which is formed by two rows of liver cells in parallel, and their branches anastomosis with each other into a network. To form a more confusing structure. The space between the liver plates forms the hepatic sinuses, which open into the central vein and are composed of endothelial cells (Figure 2b). Duck hepatocytes were polyhedral with a clear outline, round and large nuclei, most of which had a nucleus in the center of the cell. A few hepatocytes had two nuclei. The nuclear stain was light, the nuclear envelope was clear, some nucleoli were one, some were two (Figure 2c).

In silver staining, the reticular fibers were mainly distributed in the basal membrane of the central vein, interlobular arteriovenous vein, and hepatic sinuses, and the basal membrane of hepatic sinuses was discontinuous. There were no radially arranged reticular fibers around the central vein, and no obvious vascular structures between hepatocytes were found (Figure 2d–f).

In trichrome staining, it can be seen that collagen fibers are mainly distributed around blood vessels, which clearly outline of blood vessels. The central vein was also found to communicate with the interlobular vein (Figure 2g–i).

The carbon granules entering the liver during blood circulation are phagocytic by the carbon tracer technique to locate Kupffer cells (KC) in the hepatic sinuses. The results showed that there was a large number of carbon granules in the liver. The carbon granules were in the hepatic blood sinuses, and phagocytosed by Kupffer cells (Figure 2j–l).

### 3.2. Liver Lesions After Infection DTMUV and Inoculation LPS

#### 3.2.1. Anatomical Structure of the Liver After Infection DTMUV and Inoculation LPS

**Figure 3:**
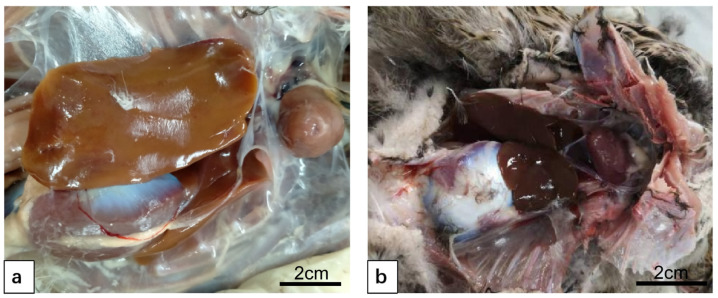
Comparison of pathoanatomical diagrams of duck liver infection with DTMUV and inoculation with LPS. (**a**) DTMUV-infected liver. The liver was found to be yellow in color, swollen and congested, tense and brittle in texture. (**b**) LPS-inoculated liver. The liver capsule was tense and slightly swollen, with no obvious change in color.

After DTMUV infection, the liver was found to be yellow in color, swollen and congested, tensed and brittle in texture (Figure 3a). After inoculation with LPS, the liver capsule was tense and slightly swollen, with no obvious change in color (Figure 3b).

#### 3.2.2. Duck Liver After Infection DTMUV and Inoculation LPS

**Figure 4:**
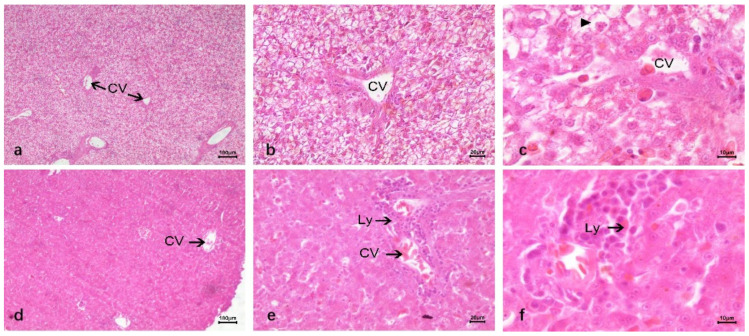
Comparison of HE staining of duck liver after infection with DTMUV and LPS. (**a**–**c**) DTMUV-infected liver. Shallow, severe vacuolar degeneration (narrow hepatic sinusoids); massive lymphocyte infiltration (clustered around central vein/portal area); increased hepatic sinus red blood cells (congestion); sinus phagocytes with abundant hemosiderin. (**d**–**f**) LPS-inoculated liver. No significant cell changes/vacuoles/substantial damage; massive lymphocyte infiltration around central vein; vascular/hepatic sinus dilatation, bleeding, increased red blood cells vs. normal; Lymphocyte (Ly); Central vein (CV); Cell vacuolization (▲).

In HE staining of duck liver infected with DTMUV, it was shallow stained and vacuolar, and the vacuolar degeneration was serious, leading to the narrow diameter of the hepatic blood sinus tube. There was a large number of lymphocyte infiltrates in the liver, and most of them clustered around the central vein and portal area. Red blood cells in the hepatic sinuses increased, and there was congestion. Phagocytic cells in the hepatic sinuses formed a large amount of hemosiderin (Figure 4a–c). Compared with DTMUV-infected duck liver tissue, LPS-inoculated liver cells showed no significant changes, no vacuole phenomenon, no substantial damage to liver cells, and a large number of lymphocyte infiltration around the central vein. There were dilatation and bleeding around blood vessels and in hepatic sinuses, and the number of red blood cells increased compared with normal duck liver tissue (Figure 4d–f).

**Figure 5:**
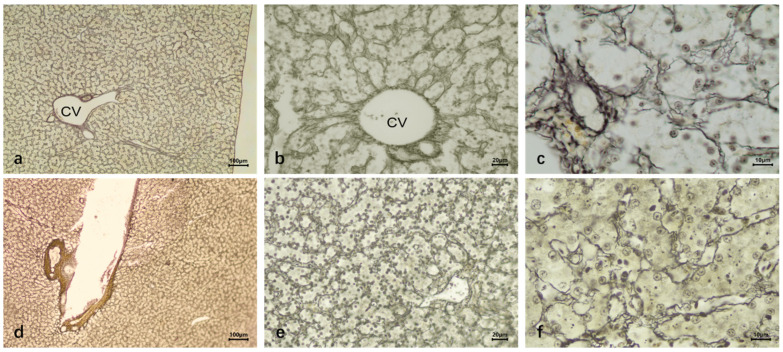
Comparison of reticular fiber staining of duck liver infected with DTMUV and inoculated with LPS. (**a**–**c**) DTMUV-infected liver. Significant increase in reticular fibers and continuous basement membrane reticular structure around hepatic sinusoids. (**d**–**f**) LPS-inoculated liver. Increase in reticular fibers; Central vein (CV).

The silver staining results of duck liver infected with DTMUV showed a significant increase in the number of reticular fibers and a continuous reticular structure of the basement membrane around the hepatic blood sinusoids (Figure 5a–c). The reticular fibers were increased in the liver tissue inoculated with LPS, even more than those after infection with DTMUV (Figure 5d–f).

**Figure 6:**
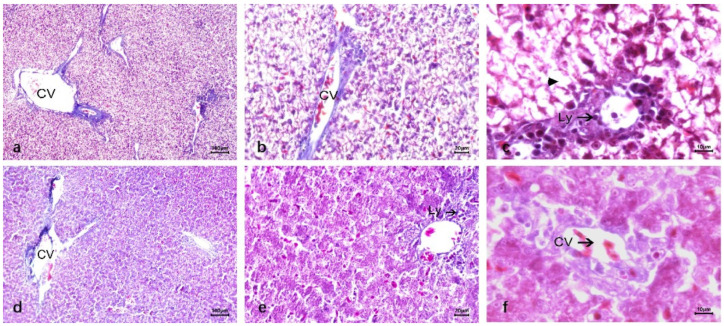
Comparison of Masson trichrome staining of duck liver after infection with DTMUV and inoculation with LPS. (**a**–**c**) DTMUV-infected liver. Trichrome staining showed portal/central vein basement membrane thickening and perivascular lymphocyte aggregation. (**d**–**f**) LPS-inoculated liver. No significant portal changes, with collagen fibers similar to normal; Lymphocyte (Ly); Central vein (CV).

Trichrome staining revealed thickening of the basement membrane of the vessels in the portal area and the central vein, and pooling of lymphocytes around the central vein and portal area (Figure 6a–c). The liver tissue inoculated with LPS showed no significant changes around the portal area, and the collagen fibers were similar to the normal liver morphology (Figure 6d–f).

**Figure 7:**
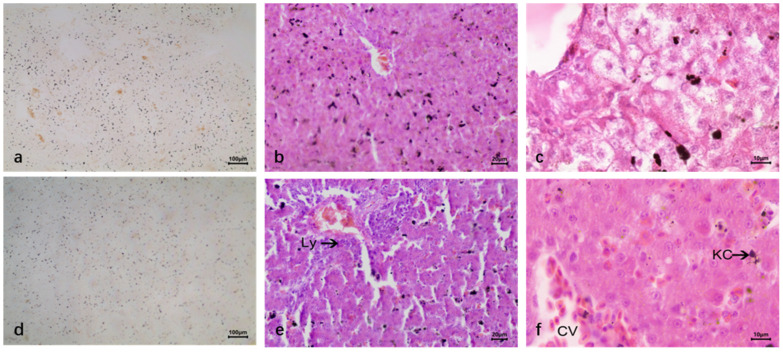
Comparison of duck liver infected with DTMUV and LPS using carbon particle tracing technology. (**a**–**c**) DTMUV-infected liver. Increased macrophage phagocytosis in the presence of blastocytes, lymphocyte infiltration, and iron-containing heme. (**d**–**f**) LPS-inoculated liver. Abundant sinusoidal carbon particles and significantly increased Kupffer cells; Central vein (CV); Kupffer cells (KC).

The carbon particle tracing technique revealed that the number of carbon particles phagocytosed by macrophages increased in the liver tissue of ducks infected with DTMUV, and the number of blastocytes in the hepatic blood sinusoids also increased, and lymphocytes infiltrated around them, and a large amount of iron-containing heme appeared in the liver (Figure 7a–c). In contrast, the liver tissue of ducks with LPS still had a large number of carbon grains and a significant increase in Kupffer cells in the hepatic blood sinusoids (Figure 7d–f).

## 4. Discussion

Among the digestive glands of animals, the liver is the largest one, and its surface is covered by a plasma membrane. In the liver, there is an extension of connective tissue at the porta hepatis, which surrounds the portal vein, hepatic artery, hepatic ducts, lymphatic vessels, and nerves, which are reached together into the liver parenchyma and divide it into several lobules [17]. If there is a lot of interlobular connective tissue, the boundaries of the lobules are very clear, as in pigs and camels; however, in cattle, sheep, horses, and birds, the interlobular connective tissue is not well developed [18], so the lobules are not clearly demarcated, and therefore the outline is not clear. Studies on the anatomy, histology, and biochemical characteristics of the liver in Pati ducks have revealed that the hepatic lobules of the liver are not obvious, due to the thin connective tissue between the lobules [19]. The distance between hepatic sinusoids and hepatocytes is closer. Therefore, nutrients and metabolic waste in the blood may exchange rapidly with the hepatocyte membrane without needing to pass through the thick connective tissue.

The hepatic lobules are composed of the central vein and hepatic plate, and sinusoids as well as hepatocytes, which are the basic structural and functional units of the liver. The arrangement of the liver plate in mammals is composed of a single row of hepatocytes, whereas in birds, in contrast to mammals, the liver plate is composed of two rows of hepatocytes, which will anastomose with each other to form a meshwork and a certain labyrinthine structure [20], and the hepatic blood sinusoids are formed by the interconnection of the liver plates, which are connected to each other by holes in the liver plate. The degree of blood filling in the hepatic blood sinusoids directly affects the morphology of the hepatic plate, which changes accordingly with the hepatic blood sinusoids. However, bile ducts, which are formed by adjacent depressions in the hepatocyte membrane, are not found visibly in the hepatic lobules of the duck liver. The portal region of the mammalian liver is composed of three parts, which are the interlobular veins, interlobular arteries, and interlobular bile ducts. The liver is the largest parenchymal organ in the body and has a complex function, with a dual pathway of portal vein and hepatic artery supplying blood to the liver, with the portal vein entering the liver, branching repeatedly to form the interlobular veins, which eventually converge into the hepatic sinusoids [21]. The remaining part of the blood comes from the hepatic artery, which branches in the interlobular connective tissue of the liver parenchyma to form the interlobular arteries and finally enters the hepatic sinusoids. Therefore, the hepatic sinusoids collect a mixture of blood from the hepatic artery and portal vein [22]. The hepatic sinusoids are a mixture of blood from the portal vein and the hepatic artery, which then branches into the sublobular veins, which in turn flow into the hepatic veins and finally into the posterior vena cava. While bile is secreted by hepatocytes, bile then enters the bile ducts, which flow from the center of the hepatic lobules to the periphery, then converge from the margins into the Herring’s duct, then to the interlobular bile ducts, which converge at the hepatic portal to the hepatic ducts out of the liver, which open to the duodenum together with the cystic ducts [23]. In this experiment, we found that no obvious interlobular bile ducts and bile ducts could be observed in adult healthy duck livers, but we could clearly observe the interlobular arteries and interlobular veins, and found that the interlobular veins are connected to the central veins. It can be inferred from this that this may be because the vascular distribution in birds is simpler than that in mammals, so there are differences in the blood circulation pathways of their livers. At the same time, the bile secreted by hepatocytes can enter the bile duct network through short-distance diffusion, while the bicarbonate and bile salts secreted by the bile ducts can also be rapidly regulated with the blood flow, and this process further improves the synergistic efficiency of digestive and metabolic processes.

The liver is mainly composed of hepatocytes, which are mainly involved in the metabolic and detoxification processes of the organism, and the remaining cells are non-parenchymal cells, including Kupffer cells and lymphocytes [24]. Kupffer cells are located in the hepatic blood sinusoids and can perform phagocytosis, engulfing and removing foreign bodies; they have a very irregular morphology and generally extend pseudopods to attach to endothelial cells, which are more evident in hepatic macrophages [25]. Carbon contouring assay was used to study the clearance capacity of phagocytes [26], and in this study, we traced the blockage of carbon particles by injected ink to duck liver blastocytes and determined their location within the hepatic blood sinusoids.

This experiment was conducted to comparatively study the phenomenon of pathological changes caused by different antigens in duck liver. Two different antigens, DTMUV and LPS, were administered to healthy duck females at the age of 4 months, and the dissection revealed different pathological changes in the duck liver caused by both. Iron-containing heme is a brownish-yellow refractive particle formed microscopically by the release of intracellular hemoglobin Fe^3+^ after phagocytosis of infected or senescent erythrocytes by macrophages. In this study, a large amount of brownish-yellow iron-containing heme was observed microscopically in the virus-infected liver, whereas iron-containing heme did not increase significantly after LPS inoculation; therefore, iron-containing heme may be responsible for the yellowish appearance of the liver.

By finding at the tissue level, the hepatocytes infected with DTMUV were severely damaged and showed obvious vacuolar degeneration. The results of optical microscopy showed a clear degeneration of the hepatic parenchymal cells in the field of view with obvious vacuolation, and it was recorded in the relevant literature that the liver of ducks infected with DTMUV showed blistering degeneration, and the present experiments presented results in the form of vacuolation [16], confirming the occurrence of blistering degeneration of hepatocytes after viral infection. The liver tissue inoculated with LPS showed insignificant cellular degeneration. It may be due to the different mechanisms of cell invasion by LPS and DTMUV [27,28]. The binding protein on LPS will bind to CD14 molecules on the surface of hepatocytes, forming a complex structure, and then, by binding to TLR4 and MD-2 molecules on the surface of the cell membrane [29,30], LPS will activate signaling pathways to transmit the desired signals into the cells, leading to the occurrence of damage in the liver [31,32]. Studies have clearly indicated that LPS triggers a series of inflammatory responses and cellular damage by activating the TLR4 signaling pathway, resulting in pathological changes in the liver of young chickens [33]. DTMUV is a single-stranded RNA virus that, upon infecting cells, activates the RIG-I/MDA5-MAVS-IRF7 signaling pathway. This leads to a significant upregulation of RIG-I and MDA5 expression, accompanied by a substantial increase in pro-inflammatory cytokines such as IL-6, IL-1β, and IL-2, ultimately resulting in cell degeneration [4,34,35]. MDA5 is involved in the innate immune response in ducks. It can recognize viral RNA and activate the interferon signaling pathway, thereby inhibiting viral replication. For example, the expression level of MDA5 is significantly upregulated after infection with the highly pathogenic H5N1 avian influenza virus [36].

This experiment also revealed that infection with DTMUV and inoculation with LPS resulted in a significant increase in the number of lymphocytes around the central vein and near the portal area. The reticular fibers in the hepatic blood sinusoids were also significantly increased, but the number of reticular fibers was higher after inoculation with LPS than after infection with DTMUV. The number of carbon particles in the hepatic blood sinusoids was significantly increased according to the carbon particle tracer localization technique, so the number of Kupffer cells increased after infection with the two different antigens. It is presumed that the two different antigens, DTMUV and LPS, stimulate the immune system in the liver. The liver, being an innate immune organ, plays an important role in resisting the invasion of pathogenic microorganisms and maintaining the normal physiological functions of the liver [37]. Numerous studies have shown that the liver is the main organ for inactivation and clearance of pathogenic microorganisms. In this experiment, female hemp ducks infected with DTMUV and inoculated with LPS at the age of four months were injected with ink, mainly to mark the Kupffer cells appearing in the liver, and compared with the control group, and the change in the number of carbon particles appearing in the liver was observed under light microscopy. Infection with DTMUV and inoculation with LPS then activated the immune system of the liver in female hemp ducks, causing an increase in the number of C. cepacia cells in the liver. Therefore, the experimental results revealed that the number of carbon grains in the tissues of the liver infected with DTMUV and inoculated with LPS was significantly increased by labeling with carbon grains, indicating that the increase in the number of Kupffer cells in the liver initiates the immune system of the organism and activates phagocytosis, which in turn leads to changes in the structure and function of hepatocytes, which is important for the systemic inflammatory response caused by different antigens.

## 5. Conclusions

Taken together, the above description shows that the normal duck liver histology is different from that of mammals. The duck liver is divided into two lobes, left and right, and the borders of liver lobules, bile duct structures, and interlobular bile ducts are not obvious, and the central and interlobular veins are connected. Infection with DTMUV and inoculation with LPS results in different pathological changes. Infection with DTMUV results in a yellow appearance, congestion, and enlargement, swelling of hepatocytes, marked vacuolar degeneration, infiltration of lymphocytes near the central vein and portal area, large amounts of iron-containing heme, and increased numbers of blastocytes, and more reticular fibers in the hepatic blood sinusoids. After inoculation with LPS, no significant degeneration of hepatocytes occurred, and there was a large collection of lymphocytes around the central vein and portal area, and the number of reticulocytes increased significantly compared to that after infection with DTMUV, as well as the number of Kupffer cells. The study of the pathological changes in the liver by comparing the two different antigens provides a theoretical basis for the pathogenicity of the avian species and the defense mechanism of the liver.

## Data Availability

The original contributions presented in this study are included in the article. Further inquiries can be directed to the corresponding author.

## Abbreviations

DTMUV	duck Tembusu virus
LPS	lipopolysaccharide
CV	Central vein
Ly	Lymphocyte
KC	Kupffer cells
